# Selective HLA Class II Allele-Restricted Activation of Atabecestat Metabolite-Specific Human T-Cells

**DOI:** 10.1021/acs.chemrestox.4c00262

**Published:** 2024-09-30

**Authors:** Megan Ford, Paul J. Thomson, Jan Snoeys, Xiaoli Meng, Dean J. Naisbitt

**Affiliations:** † Centre for Drug Safety Science, Department of Pharmacology and Therapeutics, Institute of Systems, Molecular and Integrative Biology, University of Liverpool, Liverpool L69 3GE, U.K.; ‡ Translational PK PD and Investigative Toxicology, Janssen Research & Development, Division of Janssen Pharmaceutica NV, Beerse 2340, Belgium; § AstraZeneca, The Discovery Centre, Cambridge Biomedical Campus, Cambridge CB2 0AA, U.K.

## Abstract

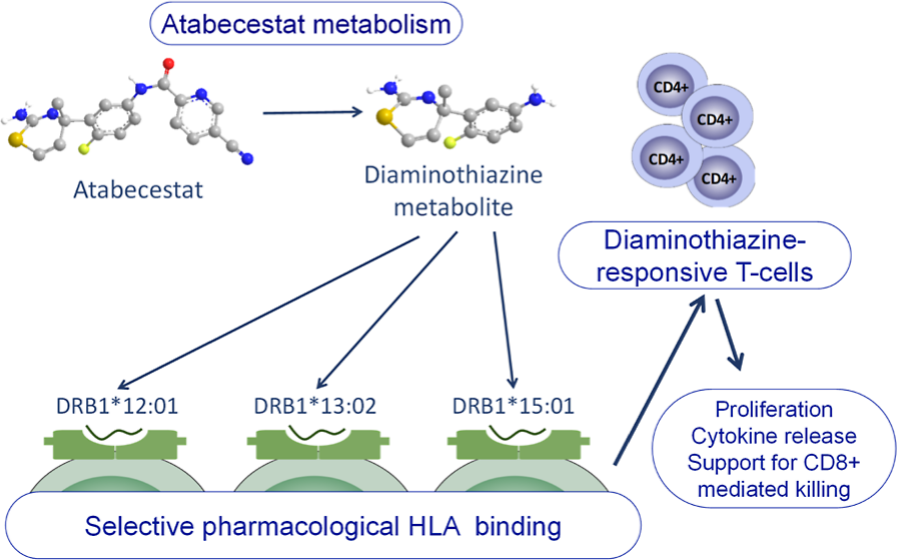

Elevations in hepatic enzymes were detected in several trial patients exposed to the Alzheimer’s drug atabecestat, which resulted in termination of the drug development program. Characterization of hepatic T-lymphocyte infiltrates and diaminothiazine (DIAT) metabolite-responsive, human leukocyte antigen (HLA)-DR-restricted, CD4+ T-lymphocytes in the blood of patients confirmed an immune pathogenesis. Patients with immune-mediated liver injury expressed a restricted panel of HLA-DRB1 alleles including HLA-DRB1*12:01, HLA-DRB1*13:02, and HLA-DRB1*15:01. Thus, the objectives of this study were to (i) generate DIAT-responsive T-cell clones from HLA-genotyped drug-naive donors, (ii) characterize pathways of DIAT-specific T-cell activation, and (iii) assess HLA allele restriction of the DIAT-specific T-cell response. Sixteen drug-naive donors expressing the HLA-DR molecules outlined above were recruited, and T-cell clones were generated. Cellular phenotype, function, and HLA-allele restriction were assessed using culture assays. Peptides displayed by HLA class II molecules in the presence and absence of atabecestat were analyzed by mass spectrometry. Several DIAT-responsive CD4+ clones, displaying no reactivity toward the parent drug, were successfully generated from donors expressing HLA-DRB1*12:01, HLA-DRB1*13:02, and HLA-DRB1*15:01 but not from other donors expressing other HLA-DRB1 alleles. T-cell clones were activated following direct binding of DIAT to HLA-DR proteins expressed on the surface of antigen presenting cells. DIAT binding did not alter the HLA-DRB1 peptide binding repertoire, indicative of a binding interaction with the HLA-associated peptide rather than with the HLA protein itself. DIAT-specific T-cell responses displayed HLA-DRB1*12:01, HLA-DRB1*13:02, and HLA-DRB1*15:01 restriction. These data demonstrate that DIAT displays a degree of selectivity toward HLA protein and associated peptides, with expression of certain alleles increasing and that of others decreasing, the likelihood that a drug-specific T-cell response develops.

## Introduction

Most drugs undergo metabolism in the liver, a process that renders the compounds more hydrophilic and excretable. For the most part, metabolism decreases the chemical reactivity and pharmacological actions of a drug through the sequential introduction of a functional group and a large conjugating agent. However, metabolism might also generate chemically reactive metabolites that bind covalently to nucleophilic amino acids within the protein. The formation of drug metabolite protein adducts is often cited as the molecular initiating event for immune-mediated adverse drug reactions targeting skin and internal organs such as liver.^1,2^ Reactive metabolites bind covalently with proteins generating adducts that stimulate immune cells via a hapten mechanism involving intracellular protein processing and the liberation of major histocompatibility complex (MHC)-associated peptide adducts.^3−5^ However, the hapten theory is not the only pathway by which T-cells are activated with drugs. Stable metabolites and the parent drug stimulate T-cells through a reversible (pharmacological) interaction with MHC or MHC-associated peptides expressed on the surface of antigen presenting cells.^6−9^ For a limited number of drugs (e.g., the pro-drug allopurinol), a stable metabolic product (i.e., oxypurinol) is thought to be the primary driver of the immune response.^8,10^

Alzheimer’s disease is a debilitating chronic condition affecting cognitive function and reduces quality of life which progresses with age. Symptoms are caused by synaptic loss, neuronal cell damage or death, and neuroinflammation in regions of the brain responsible for cognitive function. β-Site amyloid precursor protein cleaving enzyme 1 (BACE-1) is an aspartyl protease highly expressed within the nervous system that converts amyloid precursor protein into toxic peptides that can be secreted and form insoluble aggregates that deposit in amyloid plaques. These insoluble deposits lead to the loss of membrane potential, apoptosis promotion, and synaptic loss. Thus, the targeting of BACE-1 with drug inhibitors has been the focus of much Alzheimer’s disease research. Atabecestat is an orally administered BACE-1 inhibitor.^11^ Clinical trials were halted because several patients developed raised liver transaminases and fulminant liver injury.^12^ Liver enzyme elevations in patients tended to normalize with continued treatment or when atabecestat was discontinued; though in some individuals, they continued to rise after treatment was stopped. A liver biopsy performed on a single patient with ALT values 29 × the upper limit of normal, 64 days after dosing cessation, revealed an infiltration of cytotoxic lymphocytes and macrophages, suggesting that the adverse event had an immune pathogenesis.^13^ A genome-wide association study (GWAS) conducted using patients with increased liver transaminases and controls without an adverse event found that no gene variant, including human leukocyte antigen (HLA), was significantly associated with the development of liver injury. There was a suggestive association with NLRP1, SCIMP, and C1QBP, which are involved in inflammatory and immune responses.^14^ To explore the immune pathogenesis in greater detail, T-cell clones were generated from patients with atabecestat-induced liver injury and tested for drug(metabolite) specificity.^15^ T-cell clones displaying preferential responses toward a diaminothiazine (DIAT) metabolite were detected in 5 out of 8 patients using proliferation and cytokine release assays as markers of T-cell activation. Clones displayed a CD4+ phenotype, and T-cell activation was HLA-DR-restricted, with the DIAT metabolite binding directly to the HLA protein/associated peptide with no requirement for the formation of adducts or antigen processing. Recognition of DIAT by T-cells most closely resembles oxypurinol discussed above. Most clones were generated from patients expressing HLA-DRB1*12:01, HLA-DRB1*13:02, and HLA-DRB1*15:01, suggesting that responses to DIAT are restricted to individuals expressing specific HLA-DR molecules. However, no formal HLA allele restriction analysis was conducted. There is no simple explanation for the elevation of liver enzymes observed in a small number of individuals after drug discontinuation. Our working hypothesis is that drug-specific T-cells participate in the initiation of the reaction. The T-cells then cross-react with MHC-restricted peptide antigens generated naturally in the patients; however, no experimental evidence is currently available to support this.

The objective of the current study was to generate DIAT-responsive T-cell clones from healthy donors expressing the HLA-DR molecules outlined above and donors expressing unrelated HLA-DR molecules and assess the HLSA allele restriction of the drug metabolite-specific T-cell response.

## Experimental Procedures

### Cell Culture Medium

Peripheral blood mononuclear cells (PBMCs) and T-cells were cultured in R9 media made by supplementing RPMI 1640 (Sigma-Aldrich, St. Louis, MO, USA) with heat-inactivated human blood-type AB serum (5%; Innovative Research, Novi, MI, USA), l-glutamine (2 mM; Sigma-Aldrich), HEPES (4-(2-hydroxyethyl)-1-piperazineethanesulfonic acid) (25 mM; Sigma-Aldrich), transferrin (25 μg/mL; Sigma-Aldrich), and penicillin–streptomycin solution (penicillin 100 IU/ml; streptomycin 100 μg/mL; Sigma-Aldrich). IL-2 (100 IU/ml) was added to maintain the expansion of T-cell lines and clones. Immortalized Epstein–Barr virus (EBV)-transformed B-cell lines were maintained in F1 media made by supplementing RPMI 1640 with fetal bovine serum (10%; Invitrogen, Waltham, MA, USA), l-glutamine (2 mM), HEPES (25 mM), and penicillin–streptomycin solution (penicillin 100 IU/ml; streptomycin 100 μg/mL).

### Selection of Donors from a HLA-Genotyped PBMC Biobank

This study was approved by the Liverpool NHS Research Ethics Committee, and 1200 participants gave written informed consent before the study commenced. Recruitment of the volunteers was undertaken by research nurses at the University of Liverpool, and each agreed to donate 100 mL of blood. The ethical approval contained an option to recall individuals to donate additional fresh blood if required for future studies. PBMCs were isolated using density centrifugation and cryopreserved at −150 °C. HLA genotyping was then performed to obtain HLA classes I and II alleles expressed by each donor. Genotyping data obtained for the cohort is described in Alfirevic et al.^16^ and Faulkner et al.^17^

PBMCs from 16 donors expressing (HLA-DRB1*12:01 [*n* = 5], HLA-DRB1*13:02 [*n* = 3], HRA-DRB1*15:01 [*n* = 4], HLA-DRB1*13:02 and HLA-DRB1*15:01 [*n* = 1], and other HLA-DR alleles [*n* = 3]) were selected and used to establish DIAT-enriched T-cell lines prior to serial dilution. The HLA genotype of each donor is presented in Table 1.

**Table 1:**
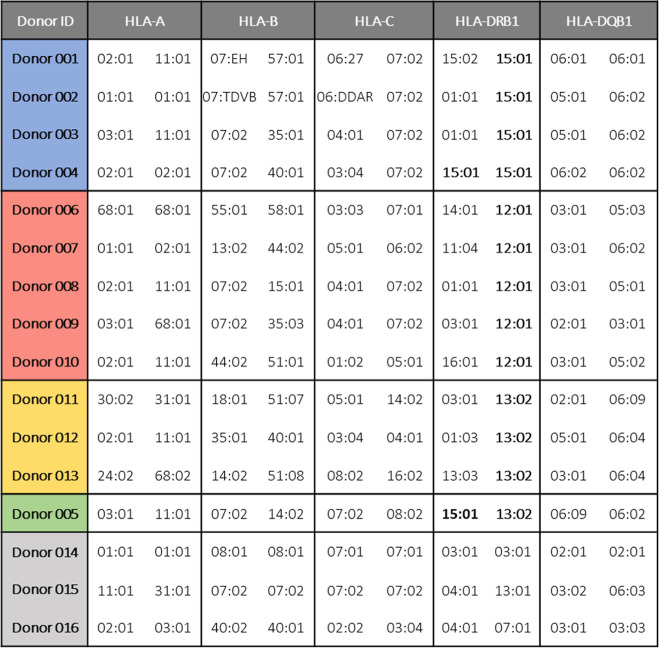
Full HLA Genotype of Healthy Donors Expressing HLA-DRB1 Alleles of Interest (Bold)^a^

a HLA-DRB1*15:01 donors are in blue, HLA-DRB1*12:01 in red, HLA-DRB1*13:02 in yellow and donors negative for these alleles in gray. Donor 5 expressing HLA-DRB1*15:01 and HLA-DRB1*13:02 is depicted in green.

### Generation of DIAT Metabolite-Enriched T-Cell Lines and Clones

Donor PBMCs (1 × 10^6^, 330 μL) were incubated in the presence of DIAT (10, 20, and 50 μM) in R9 medium (330 μL) for 2 weeks. On days 6 and 9, the PBMC medium was supplemented with IL-2 (200 IU/mL). On day 14, T-cell lines were serially diluted and subjected to phytohemagglutinin (PHA) stimulation. Briefly, T-cells (0.3–3 cells/well) were cultured with irradiated allogenic PBMC (5 × 10^4^/well) and PHA-P (1 μg/mL) in an IL-2-containing medium. The medium was supplemented with fresh IL-2 on days 6, 9, and every 2 days thereafter. Growing cultures were expanded further with a second round of mitogen stimulation and tested for drug-specific T-cell proliferation on day 28.

Immortalized autologous EBV-transformed B-cell lines were generated from healthy donor PBMCs using a supernatant from B95.8 cells. The B95.8 supernatant was filtered and mixed with PBMC (1 × 10^6^/ml) and cyclosporin A (1 μg/mL), which inhibits T-cell growth, for 16 h (37 °C, 5% CO_2_). Cells were then suspended in media (1 mL) and cyclosporin A and transferred to a 24-well plate. EBV-transformed B-cells were then maintained for 3 weeks with media containing CSA through twice weekly media supplementation. The transformed cells were used as immortalized antigen presenting cells in functional assays with T-cell clones.

Cloned T-cells (5 × 10^4^/well) were cultured with irradiated autologous EBV-transformed B-cells (1 × 10^4^/well) and DIAT in duplicate wells for 48 h. Wells containing a medium served as a negative control. Proliferation was measured by the addition of [^3^H]-thymidine, followed by scintillation counting. T-cell clones with stimulation index (SI) ≥ 2 were considered DIAT responsive and expanded using irradiated allogeneic PBMC for dose–response studies and assessment of CD4/8 phenotyping.

### Assessment of Dose-Dependent, DIAT-Specific Proliferation of T-Cell Clones and Cellular Phenotyping

Dose-dependent DIAT-specific activation of T-cell clones was performed by incubating T-cell clones (5 × 10^4^/well) with irradiated EBV-transformed B-cells (1 × 10^4^/well) and titrated concentrations of DIAT and acetyl DIAT (a major metabolite of DIAT) for 48 h in triplicate wells (37 °C, 5% CO_2_). Proliferation was measured by the addition of [^3^H]-thymidine as described above. For cellular phenotyping, T-cell clones were incubated with CD4 (clone RPA T4, APC) and CD8 (clone HIT8a, PE) fluorochrome-conjugated antibodies, respectively, on ice for 20 min. T-cell clones were washed with FACS buffer and analyzed by flow cytometry. Cells (10,000) were acquired using a FACSCanto II (BD Biosciences) and data analyzed by FACSDiva software.

### Assessment of HLA Restriction and Pathway of DIAT-Specific T-Cell Activation

To measure HLA restriction, DIAT-responsive clones (5 × 10^4^/well) and irradiated EBV-transformed B-cells (1 × 10^4^/well) were treated with anti-MHC class I (w6/32, Biolegend), MHC class II (Tu39, BD Biosciences) or HLA-DR blocking antibodies (G46–6, BD Biosciences), or isotype control IgG2a antibody (MOPC-173, Biolegend), at 10 μg/mL (37 °C, 5% CO_2_), for 2 h before addition of DIAT. After 48 h, [^3^H]thymidine was added to measure proliferation. To assess the importance of antigen processing in the DIAT-specific T-cell response, EBV-transformed B-cells were fixed with glutaraldehyde using an established method.^18^ Proliferation of T-cell clones with DIAT in the presence of fixed and irradiated EBV-transformed B-cells was then compared. Finally, EBV-transformed B-cells were pulsed with DIAT for 1–16 h to explore whether clones were activated with the DIAT-modified protein. DIAT and mock pulsed B-cells were washed with drug-free media and then cultured with clones in the absence of soluble DIAT for 48 h before addition of [^3^H]thymidine to measure proliferation.

### Assessment of HLA Allele Restriction of DIAT-Specific T-Cell Clones Using Allogenic EBV-Transformed B-Cells

Allogenic EBV-transformed B-cells were used to assess the specific HLA proteins with which DIAT interacts to activate T-cells. The donor HLA-DRB1 genotypes for the EBV-transformed B-cells selected for the HLA mismatch experiments are shown in the relevant figures. In initial experiments, T-cell clones and different EBV-transformed B-cells were incubated with the medium or DIAT, at optimum concentrations, and proliferation was measured 48 h later through addition of [^3^H]thymidine. In rare cases, donor EBV-transformed B-cells stimulated clones in the absence of drug; these cells were excluded from future experiments. Each T-cell clone was also incubated with DIAT, in the absence of EBV-transformed B-cells, to determine the degree of T-cell activation in the absence of antigen presenting cells. When T-cell proliferative responses were detected with DIAT and EBV-transformed B-cells from different donors, the experiment was repeated by using titrated concentrations of DIAT.

### Characterization of the HLA-DRB1*15:01 Peptide Binding Repertoire with and without DIAT

To elucidate the interaction between DIAT and HLA molecules, characterization of natural HLA ligands was performed using EBV-transformed B-cells from a homozygous healthy donor expressing HLA-DRB1*15:01. EBV-transformed B-cells were treated with nontoxic concentration of DIAT (50 μM) and cultured for 48 h at 37 °C, 5% CO_2_ using F1 media. Cells were pelleted, snap-frozen using liquid nitrogen, and stored at −80 °C until usage. Nondosed EBV-transformed B-cells were used as controls. Cell pellets (500,000 cells) were lysed on ice using lysis buffer containing 0.5% IGEPAL, 50 mM Tris pH 8.0, 150 mM NaCl, and protease inhibitor (complete protease inhibitor cocktail, Roche). HLA class II DR peptide complexes were isolated using anti-DR antibody (produced in-house) conjugated to PAS. HLA–peptide complexes were eluted using 10% acetic acid, as described previously. The volume of eluent was reduced in Speedvac until a volume of 500 μL. The peptides were subsequently separated from HLA heavy chain and β2-microglobulin using 5 KDa spin filters and further purified using C18 resin, dried, and stored at −20 °C for mass spectrometry analysis.

Dried samples were resuspended in 15 μL of 97% H20, 3% ACN, and 0.1% formic acid (LC–MS grade), and 1 μL of samples was injected into a TIMS quadrupole time-of-flight mass spectrometer (timsTOF Pro, Bruker Daltonics, Bremen, Germany) coupled with an ultrahigh-pressure nanoflow chromatography system (nanoElute, BrukerDaltonic). The sample was initially injected onto a trapping column (Thermo Trap Cartridge, PepMap100, C18, 300 μm × 5 mm) and then resolved on the analytical column (PepSep, 25 Series 25 cm × 150 μm × 1.5 μm column) using a gradient of 98% A (0.1% formic acid)/2% B (acetonitrile/0.1% formic acid) increasing to 65% A/35% B over 30 min, followed by a hold at 95% B for 12.5 min, at a flow rate of 0.5 μL/min. Data were acquired using the DDA-PASEF mode. Raw MS data files (.d) were processed in PEAKS Studio 11.0. Database searches were performed against the Uniprot Human proteome database Reviewed 2020 08 12 (20,356 sequences; 11,357,197 residues) and cRAP 2015 01 30 (116 sequences; 38,459 residues) contaminant database. The data were searched with the variable modification of oxidation (M; +15.99) and deamidation (NQ; +0.98), limited to 3 max variable PTM per peptide (PEAKS PTM search was also selected). Precursor mass error tolerance and fragment mass error tolerances were defined at ±25 ppm and ±0.05 Da, respectively. PSMs have been filtered to 5.0% FDR and proteins to −10 Lg *P* ≥15.0 with unique peptides ≥1. The resulting comma-separated value (CSV) files exported from PEAKS were further analyzed by R using in-house scripts.

### Statistical Analysis

GraphPad prism version 8.0 was used to perform statistical analysis. A one-way ANOVA was performed for concentration-dependent proliferation experiments if the data was normally distributed as tested by the Shapiro–Wilk test. The ANOVA analyzed multiple comparisons from the mean of the control to the mean of each concentration condition. The post hoc ANOVA test of Dunnett’s multiple comparisons was used to specify where statistical significance was found. For all other T-cell cloning assays and those that did not pass normality testing, the nonparametric *t*-test or Mann–Whitney U was performed to compare each drug treatment condition to the control.

## Results

### Generation of T-Cell Clones from Donors Expressing Different HLA-DRB1 Alleles

**Figure 1:**
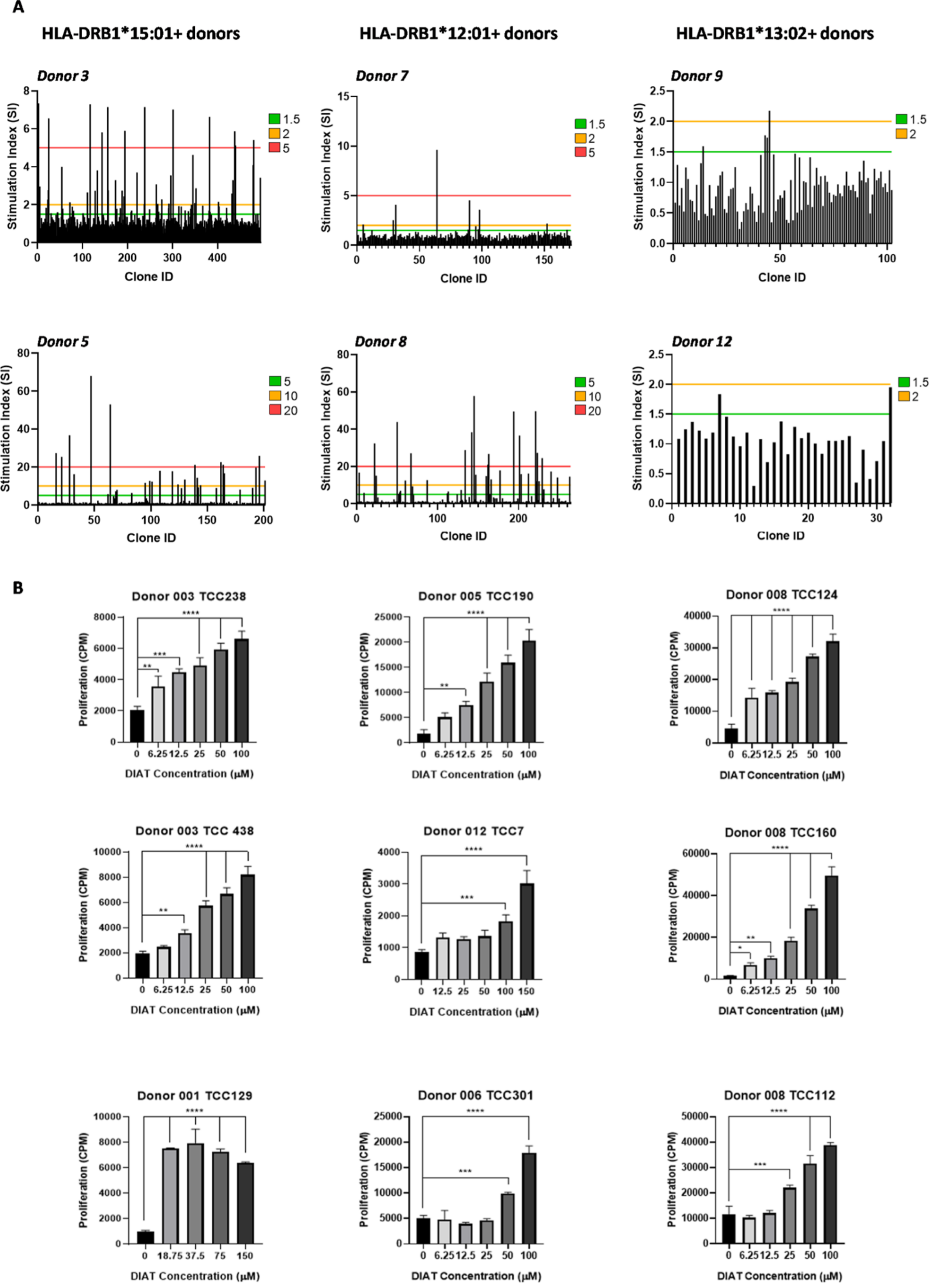
Generation of DIAT-responsive T-cell clones from drug-naïve donors expressing HLA-DRB1*12:01, HLA-DRB1*13:02, and HLA-DRB1*15:01. (A) PBMCs from 16 donors with their HLA genotype described in Table 1 were incubated for 14 days with an optimal stimulatory concentration of DIAT. PBMCs were then serially diluted to 1 cell/well in 96-U well plates and exposed to a stimulation cocktail containing IL2 (200U/mL), PHA (5 μg/mL), and irradiated allogenic PBMC (5 × 10^4^ cells/well). Well-growing single-cell cultures were then exposed to DIAT and proliferation assessed. T-cell clones (5 × 10^4^) were cultured with autologous irradiated EBV-transformed B-cells (1 × 10^4^) and DIAT (50 μM) or medium alone in duplicate for 48 h at 37 °C, 5% CO_2_. 3H-thymidine (0.5 μCi/well) was added for the last 16 h of the incubation. T-cell clones were selected for further expansion if a SI > 1.5 was demonstrated. Results from six representative donors are shown. (B) After a further 14 days, clones (5 × 10^5^ cells/well) were cultured with irradiated EBV-transformed B-cells (1 × 10^4^) and titrated concentrations of DIAT in triplicate for 48 h at 37 °C, 5% CO_2_. 3H-thymidine (0.5 μCi/well) was added for the last 16 h of the incubation to measure T-cell clone proliferation. One-way ANOVA and Dunnett’s multiple comparisons test were performed to investigate statistical significance (*p ≤ 0.05, **p ≤ 0.005, ***p < 0.001, and ****p < 0.0001). Dose–response characteristics of 9 representative clones from 6 donors are shown. DIAT-responsive clones were not detected from donors expressing alternative HLA-DRB1 alleles.

T-cell cloning was conducted at least 3 times with drug-enriched T-cell lines from donors expressing each of the 3 alleles HLA-DRB1*12:01, HLA-DRB1*13:02, and HLA-DRB1*15:01, in addition to 3 donors not expressing these alleles. In total, serial dilution experiments were conducted using DIAT-enriched T-cell lines from 16 donors. From the 2355 T-cell clones generated, 80 were deemed to be DIAT responsive upon repeat testing (Figure 1A and Table 2). Initial testing of clones involves culture of EBV-transformed B-cells with medium and an optimal DIAT concentration in duplicate. All clones displaying a SI (mean proliferation in DIAT-treated cultures/mean proliferation in medium cultures) of 2 or above were expanded and retested using titrated concentrations of DIAT in triplicate. Clones were classified as DIAT responsive if passing first test and showing proliferative responses with the drug in a dose-dependent manner.

**Table 2:** Generation of DIAT-Responsive T-Cell Clones from Donors Expressing Different HLA-DRB1 Alleles

	T-cell cloning to DIAT				
HLA-DRB1*	donor ID	tested number of clones	number of drug-responsive clones (first test)	number of drug-specific clones (repeat test)	percentage of responding clones (%)
15:01	001	176	5	1	0.6
	002	5	0	0	0
	003	495	74	9	1.8
	004	71	0	0	0
12:01	006	373	12	7	1.9
	007	171	12	4	2.34
	008	264	64	24	9.1
	009*	70	2	2	2.86
	010	50	8	4	8
13:02	011	22	3	0	0
	012	32	3	1	3.13
	013*	102	6	3	2.94
15:01/13:02	005	201	51	24	11.9
-ve	014	27	4	0	0
	015	222	4	0	0
	016	74	1	0	0

Most DIAT-responsive T-cell clones were generated from donors expressing HLA-DRB1*12:01 (*n* = 41) and HLA-DRB1*15:01 (*n* = 34). Only 4 DIAT T-cell clones were generated from 3 donors that expressed HLA-DRB1*13:02 (although donor 005 coexpressed this allele in addition to HLA-DRB1*15:01). DIAT-responsive T-cell clones were not generated from the 3 donors that did not express these HLA alleles.

**Figure 2:**
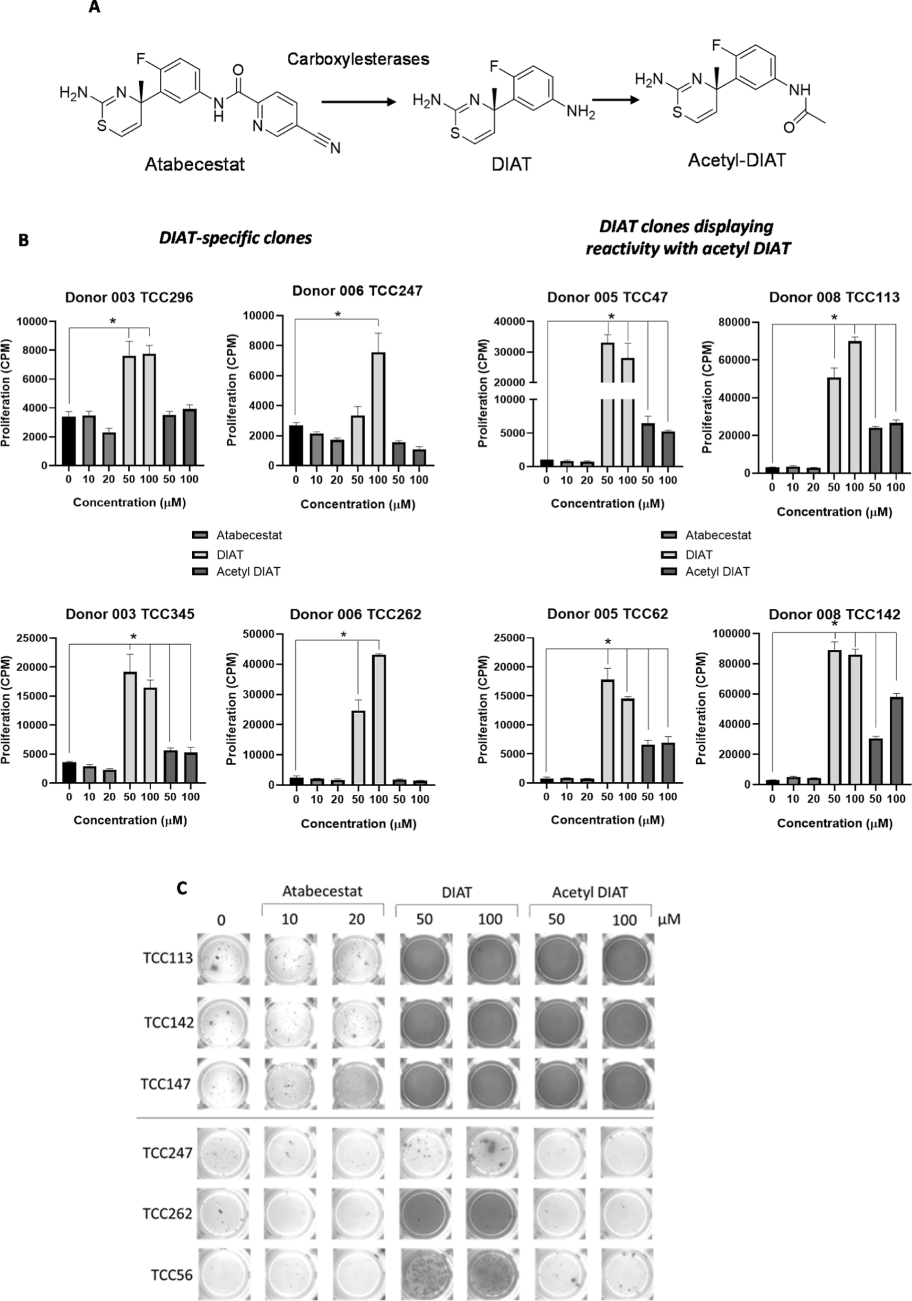
Cross-reactivity of DIAT-responsive T-cell clones. (A) Structures of atabecestat, DIAT, and acetyl DIAT. (B) T-cell clones were incubated with autologous EBV-transformed B cells in the presence of atabecestat, DIAT, or acetyl DIAT for 48 h. Medium-treated cells served as the negative control. [^3^H] thymidine was added for an additional 16 h, and proliferation was measured via scintillation counting. One-way ANOVA and Dunnett’s multiple comparisons test were performed to investigate statistical significance (*p ≤ 0.05, **p ≤ 0.005, ***p < 0.001, and ****p < 0.0001). Eight representative clones from 4 donors displaying 2 different reactivity profiles are shown. (C) IFN-γ secretion from atabecestat, DIAT, and acetyl-DIAT-stimulated T-cell clones. Clones were incubated with autologous EBV-transformed B-cells in the presence and absence of atabecestat, DIAT, and acetyl DIAT in a ELIspot plate for 48 h. Following incubation, the ELIspot was developed for IFN-γ. Six representative clones showing the 2 reactivity profiles are shown.

Clones proliferated in a concentration-dependent fashion in response to increasing concentrations of DIAT. A representative panel of 9 clones from different donors are shown in Figure 1B. Thirty-six DIAT-responsive clones from 4 donors expressing either HLA-DRB1*12:01 or HLA-DRB1*15:01 were assessed for cross-reactivity with atabecestat and a further structurally related metabolite acetyl-DIAT (Figure 2A). Nineteen clones proliferated in the presence of acetyl DIAT; however, proliferative responses were not detected against the parent drug atabecestat (Figure 2B). A similar reactivity profile was overserved when IFN-γ release was used as a marker of T-cell activation, with approximately 50% of clones displaying reactivity with DIAT and acetyl DIAT (Figure 2C). Collectively, these results indicate that (i) removal of the pyridine ring containing the reactive nitrile from atabecestat is important for the effective activation of T-cells and (ii) the MHC T-cell receptor interaction of certain clones is able to accommodate the addition of a bulky acetyl group to atabecestat. The cell surface phenotype of the T-cell clones was investigated by flow cytometry. Eighty-nine percent of the DIAT-responsive clones displayed a CD4+ phenotype; the other clones were either CD8+ or CD4+CD8+ dual positive.

### Pathway of T-Cell Activation with DIAT

**Figure 3:**
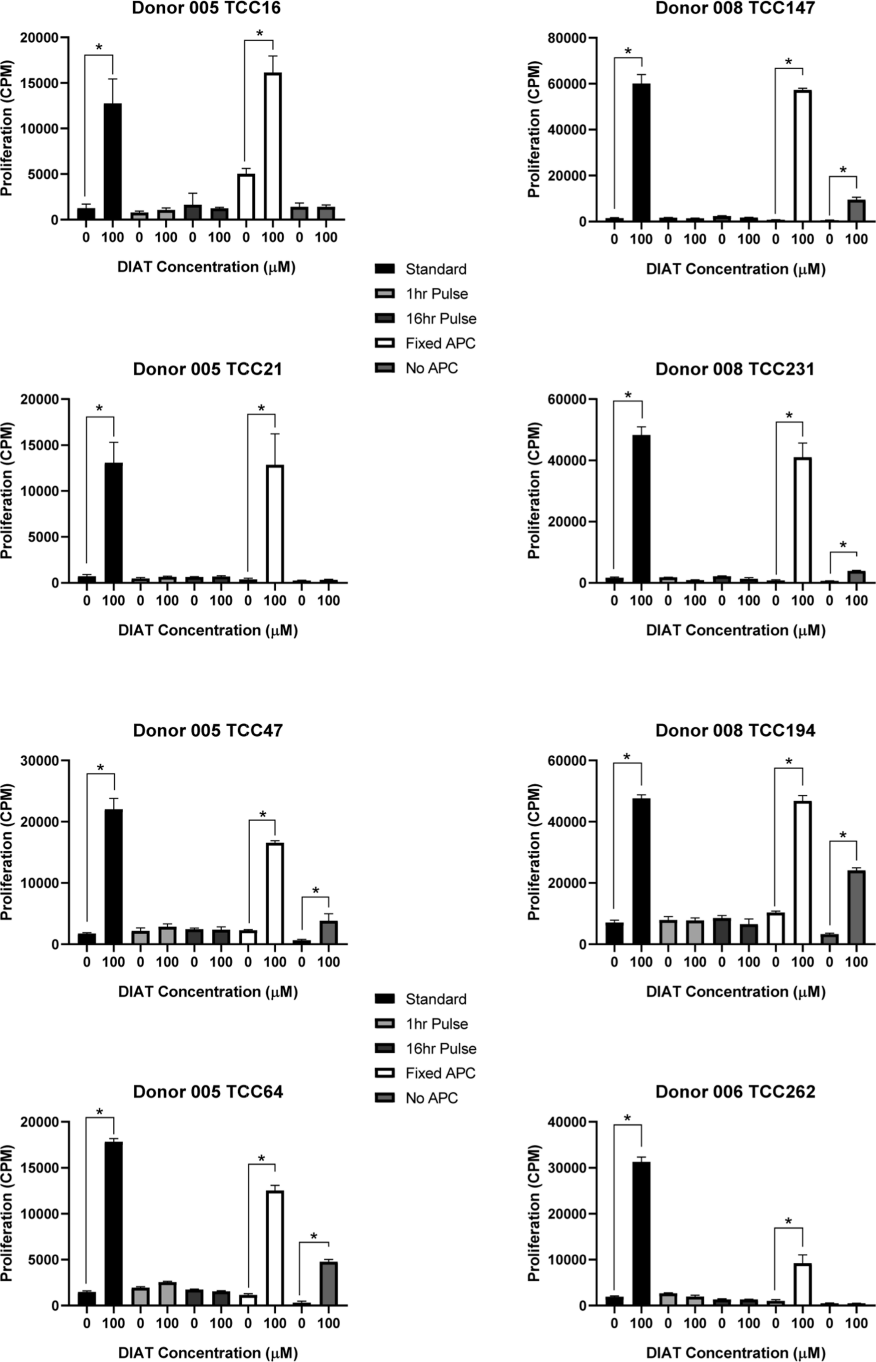
Pathway of DIAT-specific T-cell activation. T-cell clones were incubated with (i) soluble DIAT (50 μM) and irradiated autologous EBV-transformed B-cells, (ii) autologous irradiated EBV-transformed B-cells prepulsed with DIAT (50 μM) for 1 or 16 h, (iii) soluble DIAT (50 μM) and irradiated glutaraldehyde-fixed (to prevent protein processing) EBV-transformed B-cells, and (iv) soluble DIAT (50 μM) and in the absence of autologous EBV-transformed B-cells. The pulsed EBV-transformed B cells were washed repeatedly to remove free DIAT prior to culturing with T-cells. T-cell responses were measured via analysis of proliferation through addition of [^3^H] thymidine and scintillation counting. Eight representative clones from 3 donors are shown.

To determine the pathway by which DIAT-specific T-cell clones were activated, autologous EBV-transformed B-cells were incubated with DIAT (100 μM) for 1 and 16 h and then irradiated, before adding the treated cells to T-cell clones in the absence of soluble DIAT (drug haptens bind to antigen presenting cells during the pulsing period forming drug protein complexes that activate T-cells; soluble drug/metabolites are washed from the assay, and T-cell responses to a soluble material are not observed). DIAT-responsive T-cell clones were also cultured with irradiated or glutaraldehyde-fixed EBV-transformed B-cells and soluble DIAT to explore the importance of antigen processing in T-cell activation (fixation blocks antigen processing). A total of 8 DIAT-responsive T-cell clones from HLA-DRB1*15:01 and -DRB1*12:01 were studied. The clones were all stimulated to proliferate in the presence of DIAT and fixed EBV-transformed B-cells or irradiated EBV-transformed B-cells. In contrast, proliferative responses were not detected when T-cell clones were cultured with DIAT-pulsed EBV-transformed B-cells (Figure 3). Several T-cell clones were stimulated to proliferate weakly with DIAT in the absence of EBV-transformed B-cells.

### HLA Restriction of DIAT-Responsive CD4+ T-Cell Clones

**Figure 4:**
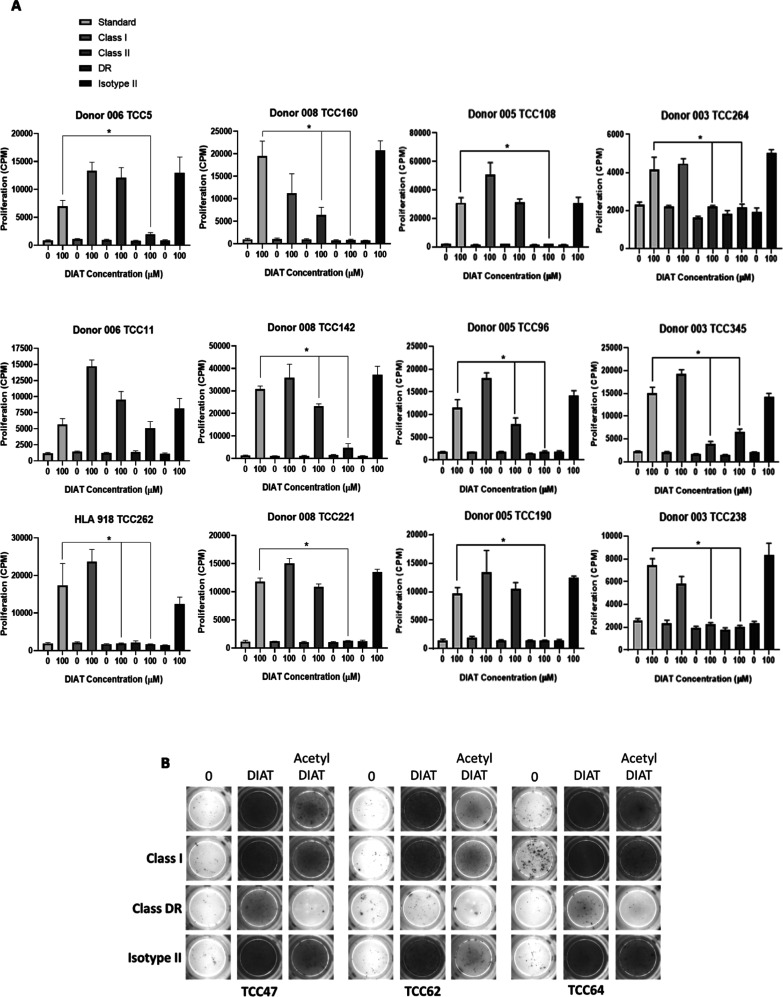
HLA-DR**-**restricted activation of DIAT-responsive T-cell clones. (A) T-cell clones (5 × 10^4^) were cultured with irradiated autologous EBV-transformed B-cells (1 × 10^4^) and either HLA class I, HLA class II, and HLA-DR (all 10 μg/mL) antibodies or corresponding IgG isotype controls (10 μg/mL) for 1 h. Blocked cultures were treated with DIAT (100 μM) or medium for 48 h before addition of [^3^H]-thymidine and scintillation counting. Nine representative clones are shown. IFN-γ secretion from DIAT and acetyl-DIAT-stimulated T-cell clones. Clones were incubated with autologous EBV-transformed B-cells in the presence of HLA class I (10 μg/mL), HLA-DR (10 μg/mL) antibodies, or corresponding IgG isotype controls (10 μg/mL) for 1 h in a ELIspot plate before addition of study compounds for 48 h. Following incubation, the ELIspot was developed for IFN-γ.

CD4+ T-cell clones (*n* = 12) and EBV-transformed B-cells were coincubated with HLA class I, HLA class II, or HLA-DR blocking antibodies before addition of DIAT to investigate HLA restriction, using proliferation and IFN-γ secretion as readouts for T-cell activation. DIAT-specific proliferation was attenuated with HLA class II block and inhibited in the presence of HLA-DR block (Figure 4A). In contrast, addition of MHC class I and isotype control antibody had no effect on the activation of clones with DIAT. Three donor 005 DIAT-responsive clones, which were cross reactive with acetyl DIAT were investigated for MHC blocking using IFNγ ELIspot. IFNγ secretion in the presence of DIAT or acetyl DIAT was inhibited in the presence of HLA class DR, but not MHC class I, block (Figure 4B).

### HLA Mismatch Experiments Using Allogenic Antigen Presenting Cells

Seventeen clones from 3 donors expressing HLA-DRB1*12:01 (donors 006 and 008) and HLA-DRB1*13:02/15:01 (donor 5) were expanded in numbers sufficient to conduct a detailed assessment of HLA allele restriction using EBV-transformed B-cells from a panel of donors expressing different HLA-DRB1 alleles. These experiments were feasible as alloreactivity (T-cell activation with nonself EBV-transformed B-cells naturally displaying peptides [in the absence of DIAT]) among clones was low.

**Figure 5:**
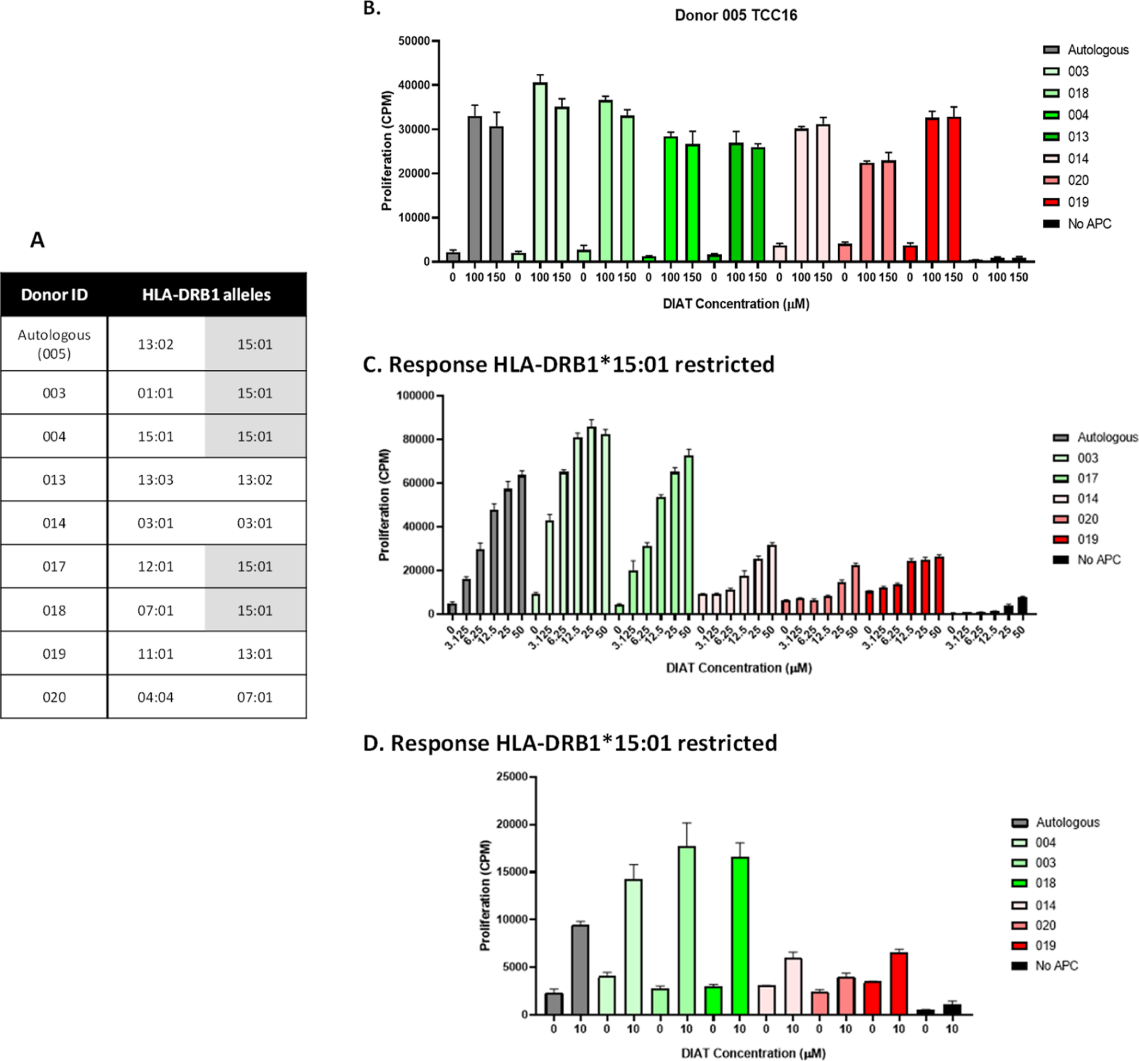
Workflow to optimize HLA mismatch assays with donor 005 TCC16 (HLA-DRB1*15:01/13:02). The HLA-DR-restricted, DIAT-responsive clone 16 (5 × 10^5^ cells/well) from donor 005 was cultured with DIAT and autologous or nonautologous irradiated EBV-transformed B-cells (1 × 10^4^) in triplicate for 48 h at 37 °C, 5% CO_2_. R9 media control was used as a negative control for each antigen presenting cell condition. Similarly, the clone was cultured with DIAT in the absence of antigen presenting cells. [^3^H] thymidine (0.5 μCi/well) was added for the last 16 h of the incubation prior to measurement of proliferation. (A) HLA-DRB1 genotype of the EBV-transformed B-cells used in the experiments. (B) Exposure of the clone to high concentrations of DIAT. (C) Exposure of the clone to lower titrated concentrations of DIAT. (D) Exposure of the clone to a low stimulatory (with autologous EBV-transformed B-cells) concentration of DIAT. Data from conditions containing EBV-transformed B-cells from donors expressing HLA-DRB1*15:01 are shown as green bars. Donors negative for the allele are shown as red bars.

Initial experiments were conducted with clone 16 from donor 005 to establish the assay conditions. Activation of the clone with optimal DIAT concentrations was dependent on the presence of EBV-transformed B-cells (Figure 5B–D), with strong, equipotent proliferative responses detected in the presence of EBV-transformed B-cells expressing alleles of interest (HLA-DRB1*13:02/15:01) and unrelated HLA alleles (Figure 5B). Hence, DIAT titration experiments were performed to determine whether DIAT displayed similar stimulatory properties when presented at suboptimal concentrations by the unrelated HLA-DRB1 molecules. In the presence of autologous EBV-transformed B-cells (donor 005) and EBV-transformed B-cells from donors expressing HLA-DRB1*15:01 (donors 003 and 017), significant DIAT-specific proliferation was observed at a concentration of 3.125 μM, and the strength of the maximal induced proliferative response was similar (50 μM; 60,000–80,000 cpm; Figure 5C). In contrast, DIAT-specific proliferation in the presence of EBV-transformed B-cells from donors expressing different HLA-DRB1 alleles (donors 014, 019, and 020) was only observed with higher concentrations (12.5–25 μM), and the maximal response at 50 μM was 20,000–30,000 cpm. Thus, DIAT displayed strong agonist properties when associated with HLA-DRB1*15:01, but weak agonistic properties when presented by different HLA-DRB1 proteins. Similar data was obtained when the experiment was repeated with 10 μM DIAT and a panel of EBV-transformed B-cells that included donor 004, which was homogeneous for HLA-DRB1*15:01 expression (Figure 5D).

**Figure 6:**
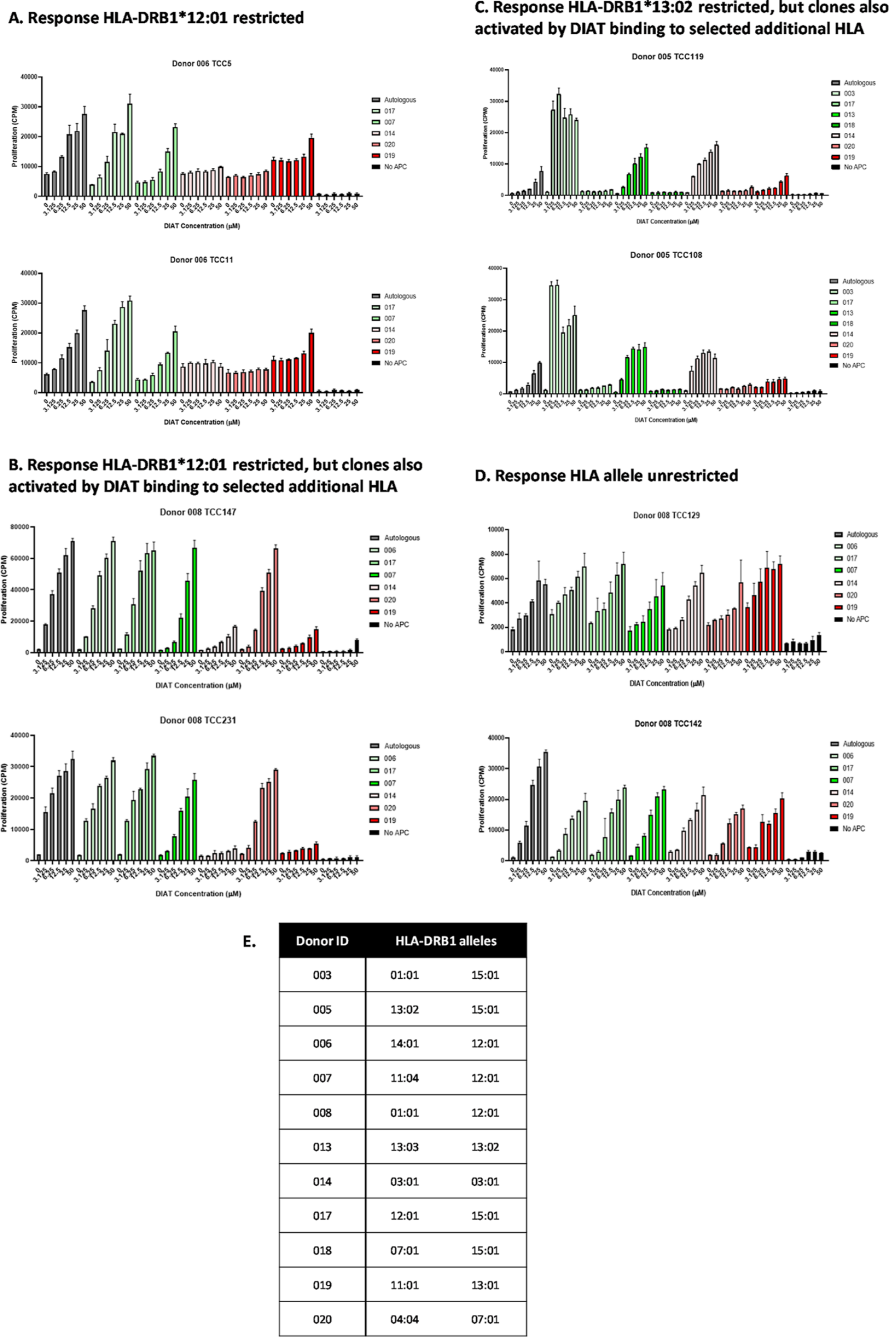
HLA-DRB1 allele restriction patterns of DIAT-responsive T-cell clones. HLA-DR-restricted, DIAT-responsive clones (5 × 10^5^ cells/well) from donors 005, 006, and 008 were cultured with titrated concentration of DIAT and autologous or nonautologous irradiated EBV-transformed B-cells (1 × 10^4^) in triplicate for 48 h at 37 °C, 5% CO_2_. R9 media control was used as a negative control for each antigen presenting cell condition. Similarly, the clone was cultured with DIAT in the absence of antigen presenting cells. [^3^H] thymidine (0.5 μCi/well) was added for the last 16 h of the incubation prior to measurement of proliferation.

Additional clones displayed 4 distinct patterns of HLA allele restriction: first, 6 clones displayed preferential proliferative responses when DIAT was presented by EBV-transformed B-cells expressing the HLA allele of interest (Figure 6A shows 2 clones from donor 006 activated with DIAT and antigen presenting cells expressing HLA-DRB1*12:01); second, 9 clones were activated with DIAT in the presence of EBV-transformed B-cells expressing the HLA allele of interest and a limited number of unrelated HLA alleles (Figure 6B shows 2 clones from donor 008 activated with DIAT and antigen presenting cells expressing HLA-DRB1*12:01 and antigen presenting cells expressing HLA-DRB1*04:04/07:01: Figure 6C shows 2 clones from donor 005 activated with DIAT and antigen presenting cells expressing HLA-DRB1*13:01/13:02 and antigen presenting cells expressing HLA-DRB1*01:01/03:01); and finally, 2 clones were activated with DIAT in the presence of EBV-transformed B-cells from all donors (Figure 6D). Interestingly, these data show that individual DIAT-responsive clones from the same donor (donor 005) are activated through a selective interaction with 2 of the HLA-DRB1 molecules (HLA-DRB1*13:02 and HLA-DRB1*15:01) identified in the atabecestat DILI patient study (15).

### DIAT HLA-DRB1*15:01 Binding Does Not Alter the Peptide Binding Repertoire

**Figure 7:**
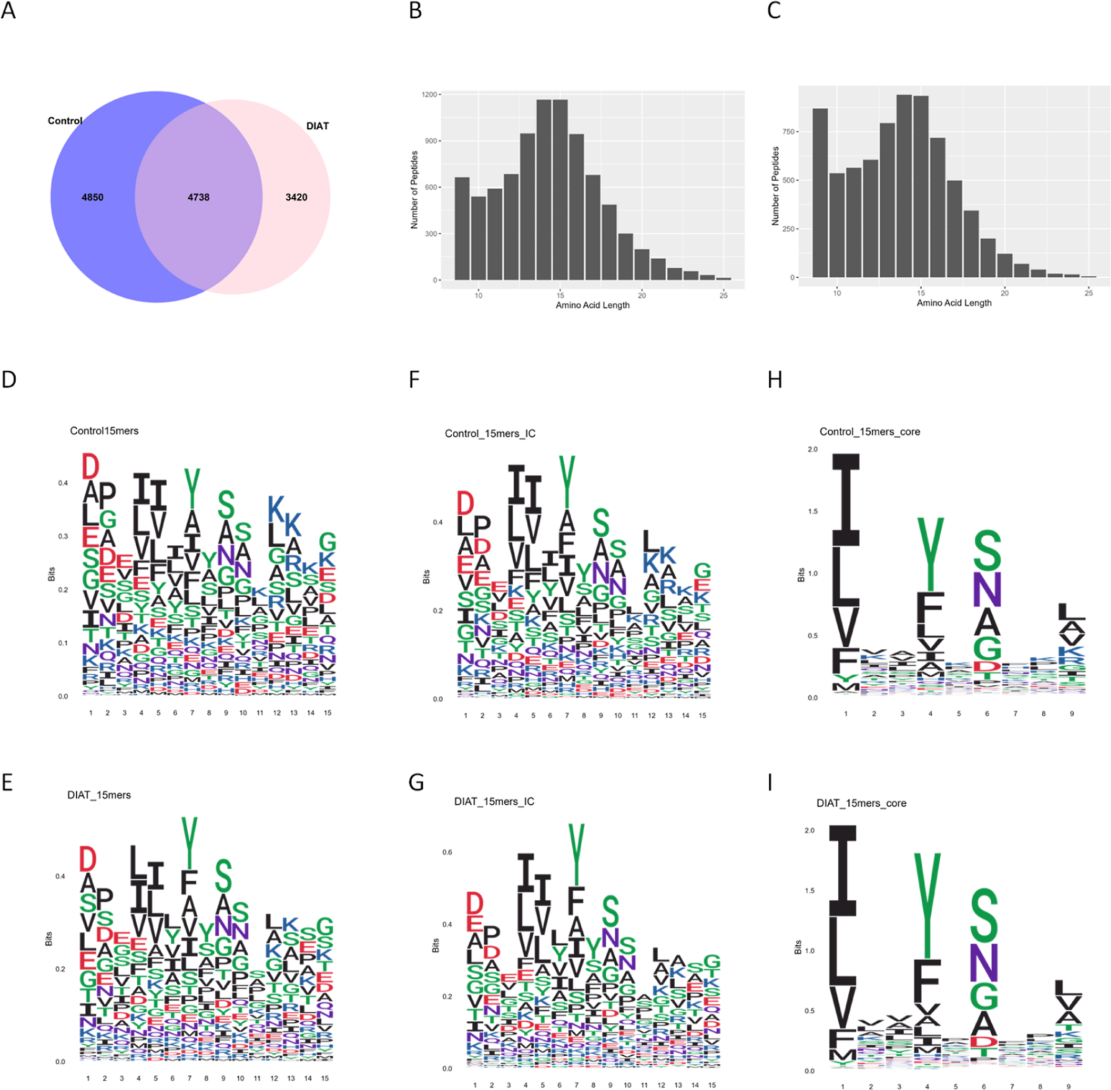
Peptide displayed by HLA-DRB1*15:01 expressed on EBV-transformed B-cells exposed to DIAT. Of the total 13,008 identified HLA-DRB1*15:01 ligandomes, 4850 peptides were exclusively identified in the untreated controls, while 3420 peptides were uniquely identified in the presence of DIAT (A). The majority of peptides were 13–17 amino acids in length in both control (B) and DIAT-treated (C). Sequence motif of all 15mers peptides shows no differences between control (D) and DIAT-treated (E). Sequence motif of HLA-DRB1*15:01 binders (predicted using the IEDB analysis resource NetMHCIIpan (ver. 4.1) tool and those with IC50 < 1000 nM were selected as binders) demonstrates enrichment of certain amino acids at P1, P4, P6, and P9 positions for control (F,H) and DIAT-treated (G,I), respectively. (Seq2Logo used to generate sequence motif graphics.)

To explore the impact of DIAT on peptide HLA-DR binding, EBV-transformed B-cells from a healthy donor with a homozygous HLA-DRB1*15:01 were incubated in the presence or absence of DIAT. HLA–peptide complexes were subsequently purified and analyzed by mass spectrometry. A total of 9588 and 8158 peptides were identified from untreated and DIAT-treated samples, respectively. 4850 peptides were exclusively identified in samples from untreated cells, whereas 3420 peptides were only identified in samples from the DIAT-treated cells. 4738 peptides were shared by both data sets (Figure 7A). For both data sets, the majority of peptides were 13–17 amino acids in length, consistent with MHC class II presentation, and no apparent changes in peptide length distribution were observed upon DIAT treatment (Figure 7B,C). To further assess whether DIAT treatment can alter position-specific preferences of amino acids in peptide sequence (peptide motif), Seq2Logo^19^ was used to generate sequence logos for 15-mer peptides. An unbiased approach without knowledge of their binding affinity to HLA-DRB1*15:01 was initially used. As demonstrated in Figure 7D,E, amino acid stacks at some positions are higher compared to other position; however, no clear pattern was observed in both data sets. The binding affinity of these 15-mer peptides to HLA-DRB1*15:01 was then predicted using the IEDB analysis resource NetMHCIIpan (ver. 4.1) tool,^20^ and those with IC50 < 1000 nM were selected as binders. Sequence logos demonstrated preference and enrichment of certain amino acid at some positions (Figure 7F,G). Further analysis of the binding cores that are predicted by NetMHCIIpan (ver. 4.1) illustrated the strong preference and enrichment of certain amino acids at P1, P4, P6, and P9 positions (Figure 7H,I), demonstrating excellent agreement with HLA-DRB1*15:01 binding motifs reported on the MHC Motif Atlas (http://mhcmotifatlas.org/).^21^ It is clear that DIAT treatment does not alter the frequency of the anchor residues, indicating that DIAT has minimal influence on HLA–peptide interaction.

## Discussion

If genome-wide association studies fail to detect an association between HLA allele expression and the development of a specific form of hypersensitivity reaction, the drug itself might still exhibit a degree of binding selectivity for and against different HLA proteins. Atabecestat may be an example of a such drug. Development of atabecestat was stopped because of an unacceptable incidence of liver injury during clinical trials.^12^ An immune pathogenesis was confirmed through detection of (i) T-cell infiltrates into inflamed liver and (ii) DIAT-responsive T-cells circulating in patient blood.^15^ Although genome-wide association studies failed to detect an association between atabecestat-induced liver injury and HLA allele expression,^14^ the DIAT-specific CD4+ T-cell response was HLA-DR-restricted, and responsive T-cells were only detected in patients carrying HLA-DR alleles such as HLA-DRB1*12:01, HLA-DRB1*13:02, and HLA-DRB1*15:01.^15^ These data led us to investigate whether (i) DIAT-responsive T-cells are preferentially detected in healthy human donors expressing these HLA-DR alleles, and if so, (ii) the drug metabolite-specific T-cell response is HLA-DR allele-restricted.

Sixteen healthy donors, 5 expressing HLA-DRB1*12:01, 3 expressing HLA-DRB1*13:02, 4 expressing HLA-DRB*15:01, and 3 expressing unrelated HLA-DRB1 alleles were selected from our HLA-typed cryopreserved PBMC bank.^16^ The final donor expressed 2 HLA-DRB1 alleles found in patients with atabecestat-induced liver injury, specifically, HLA-DRB1*13:02 and HLA-DRB1*15:01. DIAT-responsive T-cell clones were generated from donors expressing all 3 HLA-DRB1 alleles of interest. Cloning efficiency was greatest in donors 005 and 008, expressing HLA-DRB1*13:02/15:01 and HLA-DRB1*01:01/12:01, respectively. Clones were stimulated to proliferate and secrete cytokines such as IFN-γ following exposure to DIAT and autologous EBV-transformed B-cells, used as a source of autologous antigen presenting cells, with no apparent donor-to-donor difference in DIAT dose–response characteristics observed. DIAT-responsive clones were not generated from the 3 individuals expressing alternative HLA-DRB1 alleles.

T-cells are activated with drugs through the formation of a 3D complex between HLA proteins, HLA binding peptide, drug, and the T-cell receptor. A T-cell response is not detected if any one component of the complex is missing. Drug binding within the complex occurs through formation of covalent and/or noncovalent bonds.^9,18,22,23^ If a drug binds to one component of the complex covalently, other complementary noncovalent interactions must occur with other components to trigger the T-cell response. A plethora of studies have explored drug HLA protein binding interactions. Drugs or drug metabolites that contain an electrophilic moiety bind covalently to nucleophilic amino acids in the MHC binding peptide and presumably protrude from the peptide binding groove to confer antigen specificity through noncovalent binding at the T-cell receptor surface.^3,24−27^ In this form of drug-specific T-cell triggering, the drug does not interact directly with the HLA protein, although the drug–peptide interaction is likely to alter, presumably increasing the affinity of the peptide for the HLA protein. When a drug binds noncovalently, to the complex, it may mimic the actions of a hapten (e.g., carbamazepine) interacting on the outer surface of the MHC-associated peptide for recognition by the T-cell receptor.^28−31^ This pathway of drug-specific T-cell activation is readily reversible with a simple washing step removing the drug from the receptor complex, eliminating the T-cell response. Alternatively, the drug may interact directly with the MHC protein (e.g., abacavir) altering the structure of the MHC peptide binding groove and subsequently the repertoire of peptides that bind.^32−38^ Although this pathway of drug-specific T-cell activation involves noncovalent bond formation between drug and the MHC protein, the drug becomes trapped within the MHC peptide complex and is resistant to washing.

Studies with CD4+ T-cell clones from patients with atabecestat-induced liver injury demonstrated that T-cells were activated by the stable DIAT metabolite through a direct, noncovalent, binding interaction with MHC protein or the MHC-associated peptide.^15^ Herein, a similar panel of functional studies was performed with CD4+ clones from the healthy drug-naïve donors. Clones were DIAT specific, displaying no reactivity against the parent drug atabecestat. Antigen presenting cells and DIAT HLA-DR binding were prerequisites for activation of the clones; however, antigen processing and formation of a covalently bound DIAT peptide adduct were not required. To investigate whether DIAT interacts preferentially with MHC-associated peptides or the MHC protein, altering the peptide binding repertoire, MHC-associated peptides were eluted from medium and DIAT-treated EBV-transformed B-cells from a donor displaying homogeneous expression of HLA-DRB1*15:01 (donor 4) and analyzed by mass spectrometry. No difference in the HLA-DRB1*15:01 immunopeptidome was observed with DIAT treatment, indicating that the drug metabolite likely interacts with the outer surface of peptides displayed by HLA-DR proteins expressed on the surface of antigen presenting cells to stimulate a T-cell response. It is also possible that the drug interacts with the HLA protein directly above the peptide binding cleft in an orientation that permits the T-cell receptor to interact separately with the drug and peptide to confer selectivity. This seems somewhat less likely given the HLA allele restriction data discussed in detail below.

**Figure 8:**
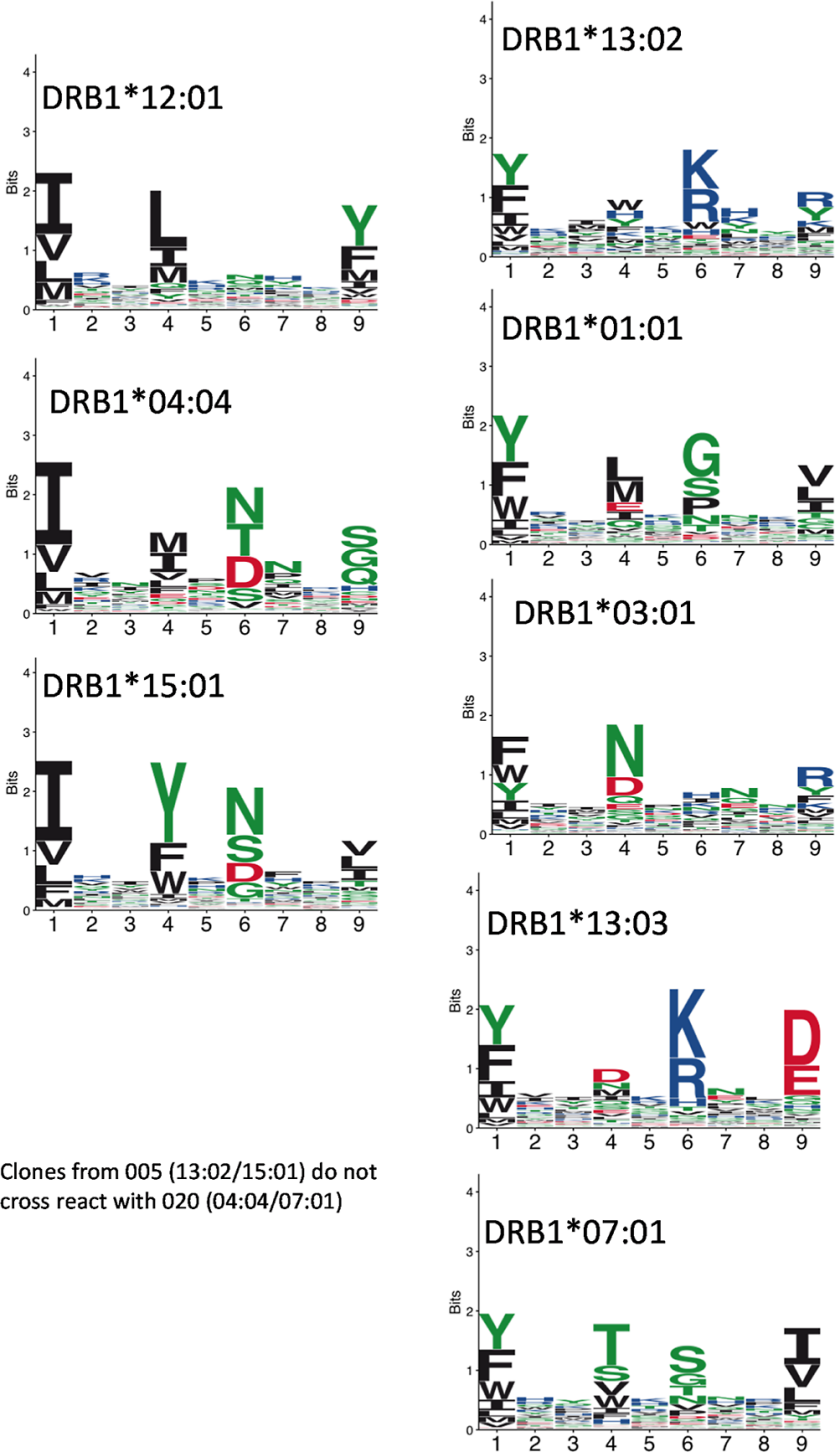
Structural comparison of HLA-DRB1*12:01, HLA-DRB1*13:02, and HLA-DRB1*15:01. HLA binding motifs of various HLA-DRB1 alleles were compared using the MHC Motif Atlas (http://mhcmotifatlas.org/). HLA class II binding motifs show distinct amino acid enrichment at positions 1, 4, 6, and 9. Whereas HLA-DRB1*12:01, HLA-DRB1*04:04, and HLA-DRB1*15:01 share the same binding motif at P1 position (I, V, and L), HLA-DRB1*13:02, HLA-DRB1*01:01, HLA-DRB1*03:01, HLA-DRB1*13:03, and HLA-DRB1*07:01 all have strong preference of Y, F, and W at P1 position.

HLA mismatch assays were performed with clones from 3 donors (donor 005, HLA-DRB1*13:02/15:01; donors 006 and 008, HLA-DRB1*12:01) to define that the HLA-DR proteins interact with DIAT to activate clones. The experiment involved incubating clones with titrated concentrations of DIAT in the presence of autologous EBV-transformed B-cells and EBV-transformed B-cells from a panel of donors expressing different HLA-DR alleles. This was feasible given the requirement of antigen presenting cells for DIAT-specific T-cell activation and the absence of alloreactivity (i.e., T-cell reactivity with peptides displayed by antigen presenting cells from the different donors). These data contrast with the work of von Greyerz et al.^39^ where cross-reactivity with allogeneic MHC molecules was substantially higher in drug-specific clones, when compared with tetanus toxoid-specific clones from the same donors. Several clones displayed clear preferential T-cell proliferative responses when exposed to DIAT and antigen presenting cells, displaying one of the three HLA-DRB1 alleles of interest. Interestingly, several clones from donor 005 were activated when DIAT interacted with either HLA-DRB1*13:02 or HLA-DRB1*15:01 (with no cross talk between the HLA-DR alleles), which illustrates that these T-cell clones receive signals from the drug and MHC protein for activation. A small number of donor 008 DIAT-responsive clones displayed an HLA-DR allele unrestricted response. With these clones, the strength of the induced proliferative response at each DIAT concentration was equipotent with antigen presenting cells expressing different HLA-DR proteins. Although drug HLA-DR binding was required for activation of the clones, the structure of the HLA-DR protein and the binding peptides was unimportant, illustrating that the dominant T-cell receptor stimulatory signal is derived from the drug. This aspect of drug-specific T-cell activation has been described previously for drugs such as lidocaine and sulfamethoxazole.^39,40^ The authors proposed two possible explanations for HLA allele-unrestricted drug-specific T-cell activation: first, the drug may bind initially to the TCR with the TCR and then receiving additional signals from the HLA peptide complex, and second, the drug may stimulate the TCR via a superantigen-like pathway. A final panel of clones were activated with DIAT in the presence of antigen presenting cells expressing the HLA allele of interest and a small number of unrelated HLA-DR proteins (e.g., donor 008 clone 231, DIAT interacted with HLA-DRB1*12:01 and HLA-DRB1*04:04 or 07:01; donor 005 clone 108, DIAT interacted with HLA-DRB1*13:02 and HLA-DRB1*01:01 or 03:01). This panel of reactivity could not be explained simply through HLA allele alignment conducted using the IDP-MHC multiple sequence alignment tool. Amino acid sequences overlapped when DIAT interacting and noninteracting HLA-DR proteins were compared, and there was no clear structural similarity with the DIAT interacting proteins. Next, HLA binding motifs of HLA-DRB1*12:01, HLA-DRB1*13:02, and HLA-DRB1*15:01 were compared using the MHC Motif Atlas (http://mhcmotifatlas.org/) (Figure 8). HLA class II binding motifs show distinct pockets at positions 1, 4, 6, and 9 within the binding cleft which are responsible for peptide binding specificity.^41,42^ Some similarity at P1 was observed between HLA-DRB1*12:01, HLA-DRB1*04:04, and HLA-DRB1*15:01, all showing preference of I, V, and L. Likewise, HLA-DRB1*13:02, HLA-DRB1*01:01, HLA-DRB1*03:01, HLA-DRB1*13:03, and HLA-DRB1*07:01 all have strong preference of Y, F, and W at P1 position. This indicates that DIAT could potentially interact with these anchor residues, leading to T-cell activation. The T-cell receptor clonotypes of DIAT responsive clones are being investigated in ongoing experiments to determine whether this helps to explain the HLA-DRB1 restriction patterns.

As described above, previous studies have shown that drugs which activate T-cells through a direct HLA peptide/T-cell receptor interaction (e.g., lidocaine and sulfamethoxazole) do so via an antigen-presenting cell-dependent, but HLA-DR allele-independent manner.^40,43−45^ A similar pathway of T-cell activation can be seen with a small number of DIAT-responsive clones; however, this seems to be the exception rather than the rule. Our data showing that (i) the preferential generation of DIAT-responsive clones from donors expressing HLA-DRB1*12:01, HLA-DRB1*13:02, and HLA-DRB1*15:01 and (ii) HLA-DRB1 allele-restricted T-cell activation demonstrates a clear specificity in the drug immune receptor binding interaction. Therefore, even when genome-wide association studies do not detect a relationship between HLA allele expression and the development of an adverse event, the culprit drug may display a complex HLA protein/peptide binding interaction, with the expression of certain alleles increasing and others decreasing the likelihood that a drug-specific CD4+ T-cell response develops. Activation of CD4+ T-cells results in a proliferative response and secretion of cytokines such as IFN-γ, which supports the recruitment of macrophages and cytotoxic T-cells. The different components of the immune system presumably work together to bring about tissue injury observed in patients with liver injury.

## Abbreviations

PBMC: peripheral blood mononuclear cells
SI: stimulation index
EBV: Epstein–Barr virus
MHC: major histocompatibility complex
HLA: human leukocyte antigen
GWAS: genome-wide association study
BACE-1: beta-site amyloid precursor protein cleaving enzyme 1
